# Total pancreatectomy with remnant stomach preservation in a patient with a history of proximal gastrectomy and interposed jejunal reconstruction with right gastroepiploic conduit preservation: a case report

**DOI:** 10.1186/s40792-023-01700-0

**Published:** 2023-06-25

**Authors:** Masaru Matsumura, Masahiro Kobayashi, Satoshi Okubo, Shusuke Haruta, Rikako Koyama, Hironori Uruga, Junichi Shindoh, Tsunao Imamura, Yutaka Takazawa, Masaji Hashimoto

**Affiliations:** 1 Hepato-Biliary-Pancreatic Surgery Division, Department of Gastroenterological Surgery, Toranomon Hospital, 2-2-2 Toranomon, Minato-Ku, Tokyo, 105-8470 Japan; 2 Department of Gastroenterological Surgery, Toranomon Hospital, Tokyo, Japan; 3 Department of Gastroenterology, Toranomon Hospital, Tokyo, Japan; 4 Department of Diagnostic Pathology, Toranomon Hospital, Tokyo, Japan; 5 Okinaka Memorial Institute for Medical Research, Tokyo, Japan

**Keywords:** Pancreatic resection, Proximal gastrectomy, Right gastroepiploic artery, Right gastroepiploic vein

## Abstract

**Background:**

Pancreatic head resection following proximal gastrectomy jeopardizes the blood flow of the remnant stomach owing to right gastroepiploic conduit sacrifice, thereby necessitating total gastrectomy. However, owing to its high invasiveness, concomitant remnant total gastrectomy with pancreatectomy should be avoided as much as possible. Herein, we describe our experience of total pancreatectomy with right gastroepiploic conduit preservation in a patient with a history of proximal gastrectomy and reconstruction by jejunum interposition.

**Case presentation:**

A 78-year-old woman with a history of gastric cancer was followed up at our institute for multiple intraductal papillary mucinous neoplasm, and main pancreatic duct stricture in the pancreatic head was newly detected. The cystic lesion was extended to the pancreatic body. Proximal gastrectomy and reconstruction by jejunal interposition were previously performed, and the mesenteric stalk of the interposed jejunum was approached through the retrocolic route. We planned total pancreatectomy with right gastroepiploic conduit preservation. Following adhesiolysis, the interposed jejunum and its mesentery lying in front of the pancreas were isolated. The arterial arcade from the common hepatic artery to the right gastroepiploic artery was detached from the pancreas. Furthermore, the right gastroepiploic vein was isolated from the pancreas. The pancreatic body and tail were pulled up in front of the remnant stomach, and the splenic artery and vein were resected. The pancreatic body and tail were pulled out to the right side, and the pancreatic head was divided from the pancreatic nerve plexus to the portal vein. The jejunal limb for entero-biliary anastomosis was passed through the hole behind the superior mesenteric artery and vein, and gastrointestinal anastomosis using the antecolic route and Braun anastomosis were performed.

**Conclusions:**

To avoid remnant total gastrectomy, right gastroepiploic conduit preservation is an optional procedure for pancreatic head resection in patients who have undergone proximal gastrectomy with reconstruction by jejunal interposition.

## Background

Pancreatic head resection requires sacrifice of the right gastroepiploic artery (RGEA) and vein (RGEV) [1]. Although they are one of the main feeders and drainage routes of the stomach, they can be resected in gastric surgery because the stomach has several blood routes other than these vessels. However, the right gastroepiploic conduit plays a significant role in a remnant stomach in patients with a history of proximal gastrectomy, and right epiploic conduit sacrifice jeopardizes the blood flow of the remnant stomach, thereby necessitating total gastrectomy. However, total gastrectomy is frequently avoided even for gastric cancer because it reduces postoperative nutritional status and quality of life [2–4]. Moreover, reconstruction following pancreatectomy and a concomitant gastrectomy in a patient with a history of proximal gastrectomy is complex. Therefore, remnant total gastrectomy should be avoided as much as possible in patients who undergo pancreatic head resection following proximal gastrectomy. As a type of modified surgery, several reports have discussed pancreatoduodenectomy with right gastroepiploic conduit preservation [5–23]. Herein, we describe our experience of total pancreatectomy with right gastroepiploic conduit preservation in a patient with a history of proximal gastrectomy and reconstruction by jejunum interposition.

## Case presentation

**Fig. 1:**
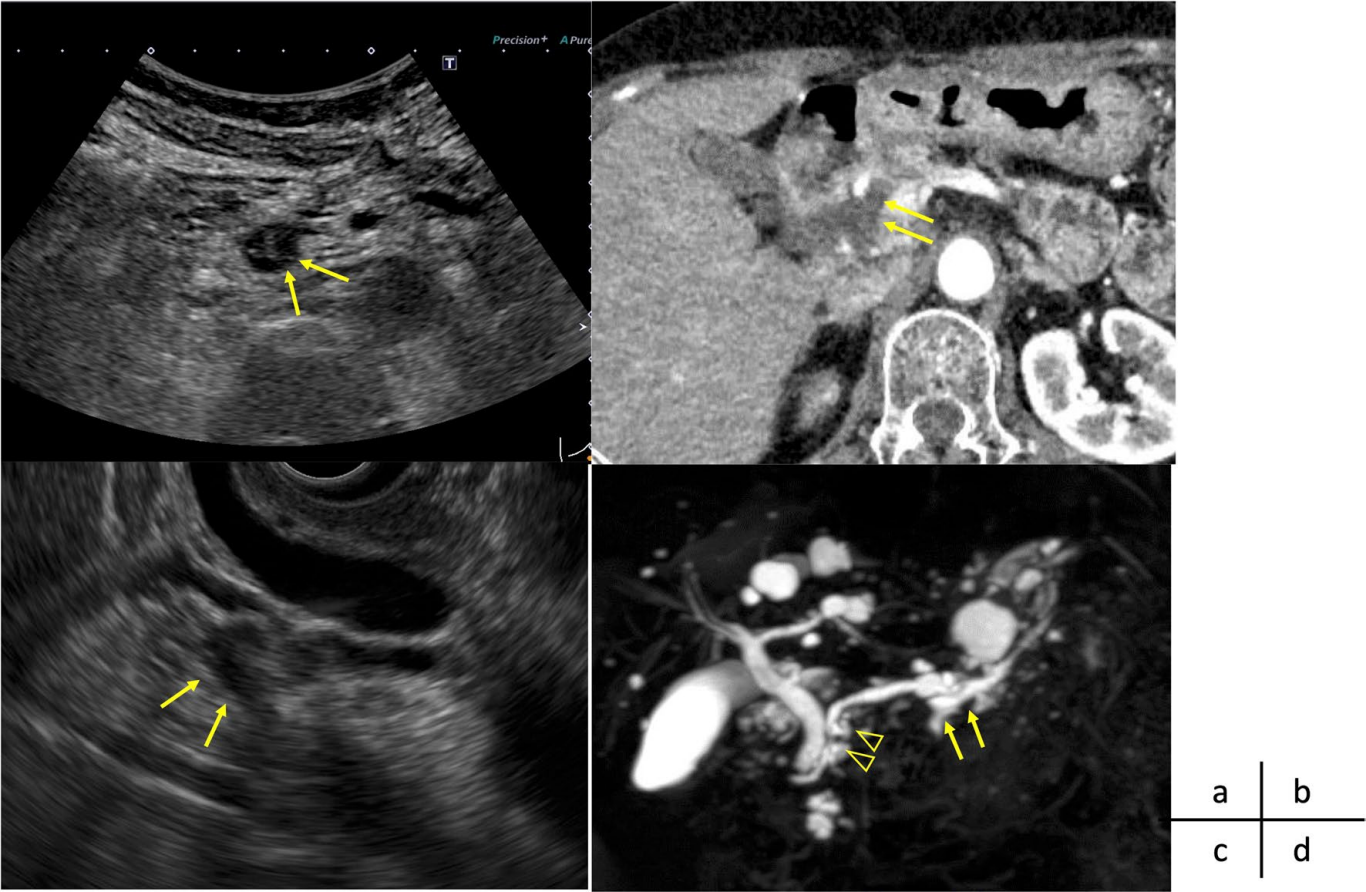
Preoperative imaging findings. **a** Abdominal ultrasonography. An intraluminal nodule is suspected in the cystic lesion at the uncinate process (arrow). **b** Computed tomography in the arterial phase. A 16-mm-diameter cystic lesion is detected in the uncinate process (arrow), and a solid portion in the cyst is not evident. **c** Endoscopic ultrasonography. The cystic lesion shows a thickened wall, and the intramural echoic shadow is unclear; however, intramural nodules and mass are not detected (arrow). **d** Magnetic resonance cholangiography. Main pancreatic duct stricture (triangle) at the pancreatic duct is newly detected. The cystic lesion in the pancreatic body has not changed since the previous imaging study (arrow)

A 78-year-old woman was followed up at our institute for multiple intraductal papillary mucinous neoplasm (IPMN). She underwent proximal gastrectomy for gastric cancer 20 years ago. During follow-up for IPMN, ultrasonography revealed a cystic lesion at the uncinate process that enlarged from 15 to 16 mm in diameter raising suspicion of intramural nodules. Enhanced computed tomography revealed that the cystic lesion at the uncinate process was 16 mm in diameter, and a solid portion of the cyst was not evident. Endoscopic ultrasonography (EUS) revealed thickened walls of the cystic lesion and an unclear intramural echoic shadow; however, intramural nodules and mass were not detected. The main pancreatic duct was 6 mm in diameter, and the carbohydrate antigen (CA) 19-9 level was 112 U/L. The patient was diagnosed with branched-type IPMN with worrisome features, including an increased CA 19-9 level and a dilated main pancreatic duct; follow-up examination was scheduled shortly thereafter [24]. Magnetic resonance cholangiography performed 3 months following EUS revealed main pancreatic duct stricture in the pancreatic head. The cystic lesion in the pancreatic body was unchanged since the previous imaging study (Fig. 1d). The CA 19-9 level increased to 172 U/L. Based on these findings, IPMN with main pancreatic duct involvement was suspected, and surgical resection was planned. The patient did not have glucose intolerance and did not use insulin.

**Fig. 2:**
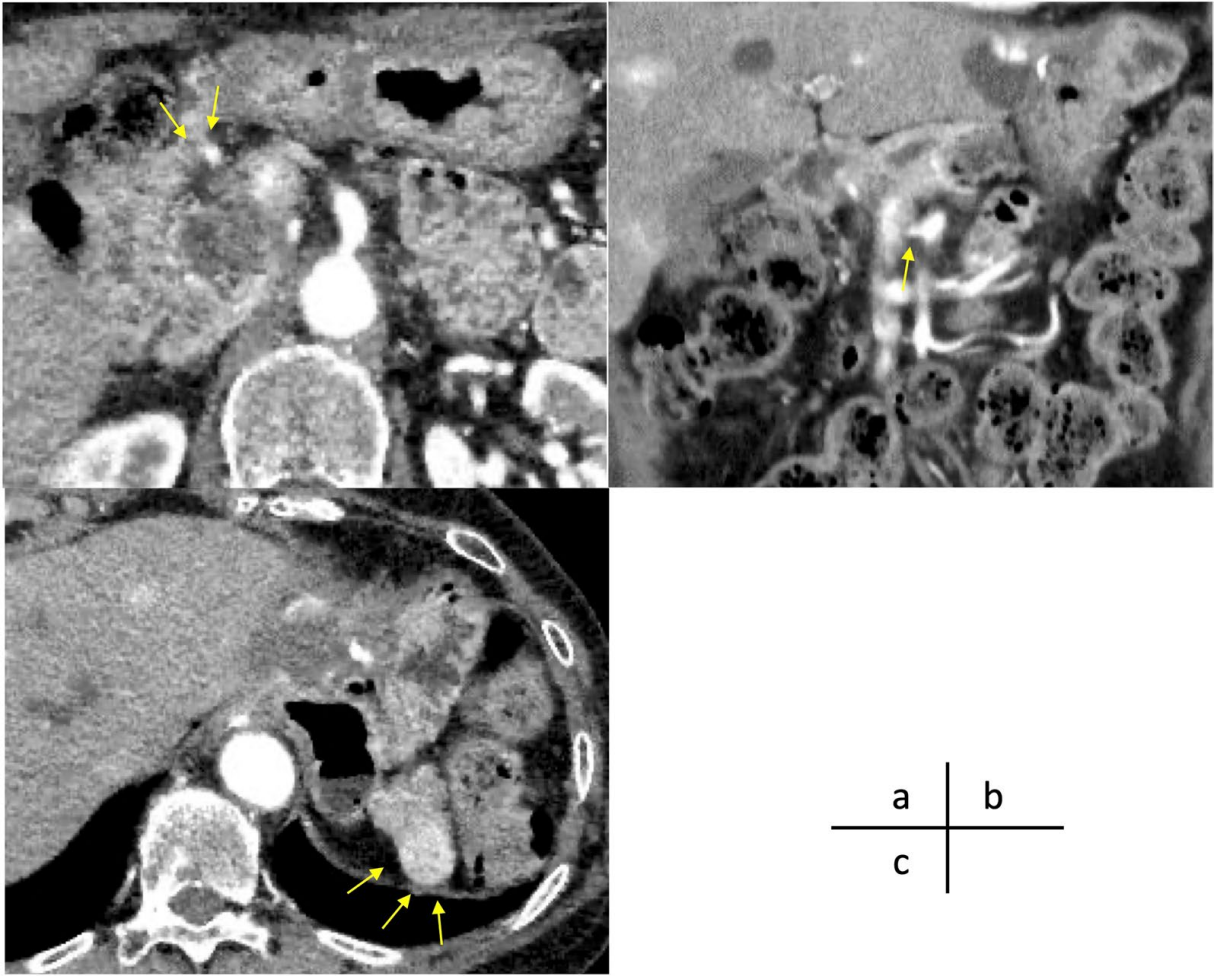
Preoperative study of computed tomography in the arterial phase. **a** The gastroduodenal artery (GDA) ran the surface of the pancreas (arrow). **b** The right hepatic artery branches from the superior mesenteric artery. **c** The accessory spleen at the pancreatic tail (arrow)

Although the lesion suspected of malignancy was confined to the pancreatic head, the pancreatic body showed involvement of a moderately large cystic lesion, and resection involving the pancreatic body was considered reasonable. However, resecting both pancreatic head and body would result in a very small pancreatic tail, making anastomoses difficult; therefore, total pancreatectomy was considered. The operative record of the previous gastric surgery showed that the surgery performed was proximal gastrectomy with splenectomy and lymph node dissection around the pancreatic tail and reconstruction with jejunal interposition. The mesenteric stalk of the interposed jejunum was approached through the retrocolic route. Regarding anatomy, the gastroduodenal artery (GDA) ran along the surface of the pancreas. The right hepatic artery (RHA) branched from the superior mesenteric artery (SMA) and ran behind the pancreatic head. The RGEV entered into the superior mesenteric vein (SMV), merging with drainage veins from the pancreatic head. The gastrocolic trunk was not formed, and the accessory right colic vein was absent. The left gastric artery and vein were resected, and the right gastric artery was not clearly visualized. A 2-cm-diameter mass was observed in the pancreatic tail, which was compatible with an enlarged accessory spleen following splenectomy (Fig. 2).

To reduce the surgical invasiveness and influence on the postoperative nutritional status, we considered pancreatectomy with remnant stomach preservation. The right gastric and right gastroepiploic conduits were the remnant vessels to the stomach, whereas the flow of the right gastric artery seemed poor. We intended to avoid ischemia or congestion of the remnant stomach; therefore, maintaining the flow of the RGEA and RGEV was essential. No evidence of any invasive features of the neoplasm to the right gastroepiploic conduit was noted; therefore, total pancreatectomy with right gastroepiploic conduit preservation was planned.

**Fig. 3:**
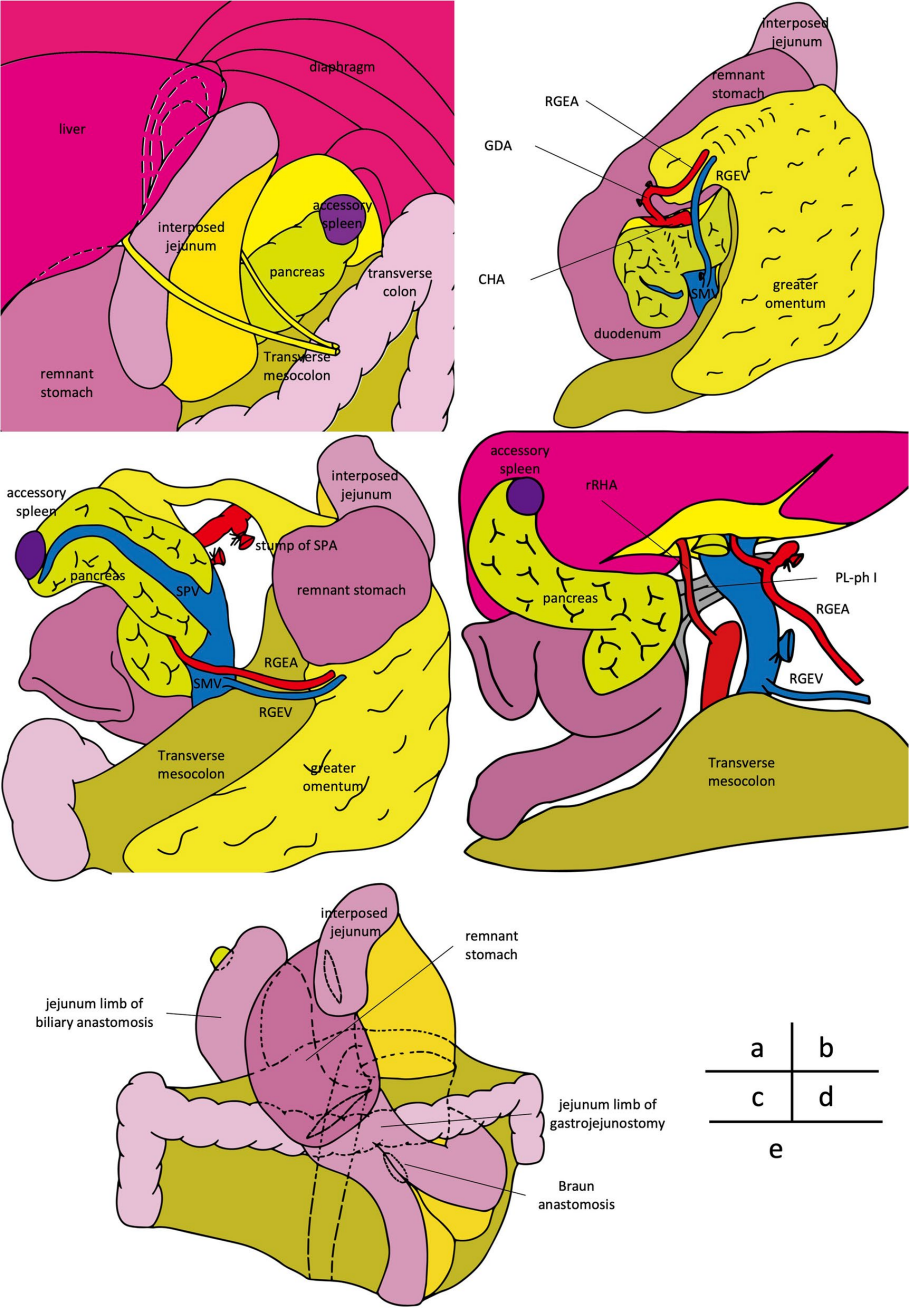
The scheme of intraoperative findings. **a** Taping of the interposed jejunum. The edge of pancreatic tail is exposed at the left subphrenic portion, and the interposed jejunum and its mesentery lying in front of the pancreas are isolated and taped. **b** Isolation of the right gastroepiploic conduit. The GDA is divided from the pancreas, and the arcade from the common hepatic artery (CHA) to the right gastroepiploic artery (RGEA) is isolated. The right gastroepiploic vein (RGEV) from the superior mesenteric vein (SMV) is isolated from the pancreas by resecting the branches to the pancreas. **c** Resection of the splenic artery (SPA) and vein (SPV). By guiding the CHA, the SPA is isolated and resected at the root. The pancreatic body and tail are turned up in front of the remnant stomach, and the root of the SPV is resected. **d** The resected jejunum is pulled out to the right side, and resection around the superior mesenteric artery plexus is performed with preserving the right hepatic artery (RHA) from the SMA. Finally, the pancreatic head is divided from the pancreatic nerve plexus I (PL-ph I), and the surgical specimen is removed. **f** Reconstruction route of the jejunum. The jejunal limb for entero-biliary anastomosis is passed through the hole behind the SMA and SMV (retromesenteric route). The jejunum for gastrointestinal anastomosis using the antecolic route and the Braun anastomosis are created. *RGEA* right gastroepiploic artery, *RGEV* right gastroepiploic vein, *GDA* gastroduodenal artery, *CHA* common hepatic artery, *SMV* superior mesenteric vein, *SPA* splenic artery, *SPV* splenic vein, *RHA* replaced right hepatic artery, *PL-ph I* pancreatic nerve plexus I

**Fig. 4:**
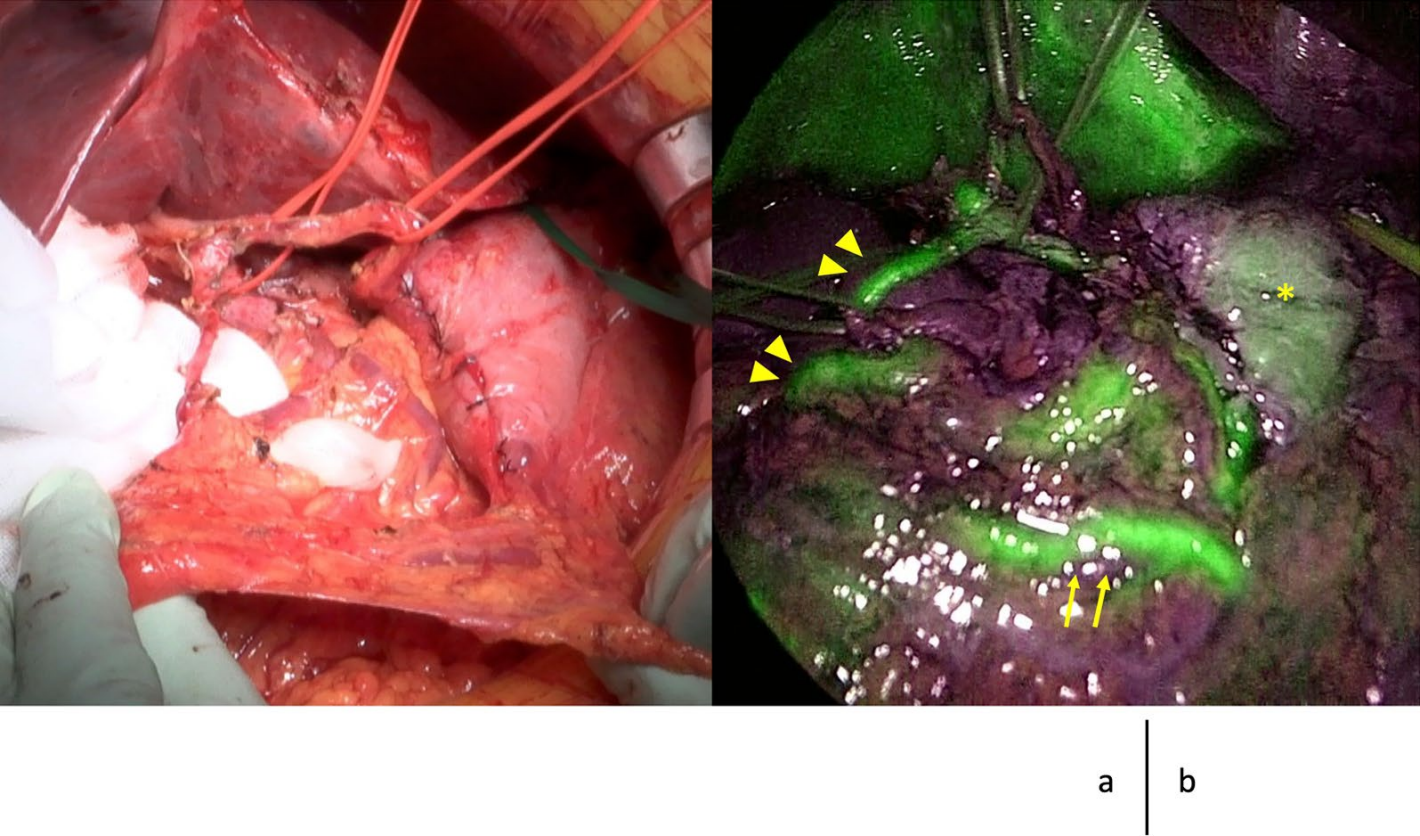
Intraoperative images before reconstruction. **a** Original image. **b** Fluorescent image. Fluorescent imaging with 7.5 mg of intravenous indocyanine green clearly visualizing the flow of the right gastroepiploic artery, (triangle), right gastroepiploic vein (arrow), and the remnant stomach (*)

A midline incision was made. Following adhesiolysis, the interposed jejunum and its mesentery lying in front of the pancreas tail was isolated (Fig. 3a). The RGEA was isolated near the gastric wall, and the GDA running along the surface of the pancreas was detected following the RGEA and isolated from the pancreas. Thereafter, the SMV was exposed and the RGEV was also isolated from the pancreas by resecting the branches to the pancreas (Fig. 3b). The remnant stomach was dissected using a linear stapler 5 cm proximal to the pylorus without resecting the right gastric conduit pedicle. The common hepatic artery (CHA) was isolated, and the arterial arcade from the CHA to the RGEA was detached from the pancreas. Lymph node dissection around hepatoduodenal ligament was performed. Pancreatic body and tail were pulled up in front of the remnant stomach, and the splenic artery was isolated by guiding the CHA and resecting at the root. The pancreatic body was turned up, and the root of the splenic vein was resected (Fig. 3c). The proximal portion of the jejunum was resected from 15 cm at the beginning of the jejunum, and the mesenteric membrane was resected near the intestinal wall avoiding injury to the marginal arcade for the interposed jejunum. The resected jejunum, pancreatic body, and tail were pulled out to the right side, and the pancreatic head was divided from the pancreatic nerve plexus and portal vein. Here, the replaced RHA from the SMA was preserved, and the surgical specimen was removed (Fig. 3d). Fluorescent imaging with 7.5 mg of intravenous indocyanine green clearly revealed the flow of the RGEA, RGEV, interposed jejunum, and remnant stomach (Fig. 4). Because the retrocolic route was crossed with the mesenteric stalk of the interposed jejunum and the blood flow of the jejunum might have been jeopardized, the jejunal limb for entero-biliary anastomosis was passed through the hole behind the SMA and SMV (retromesenteric route). Following entero-biliary anastomosis, gastrointestinal anastomosis using antecolic route and Braun anastomosis were performed (Fig. 3e).

**Fig. 5:**
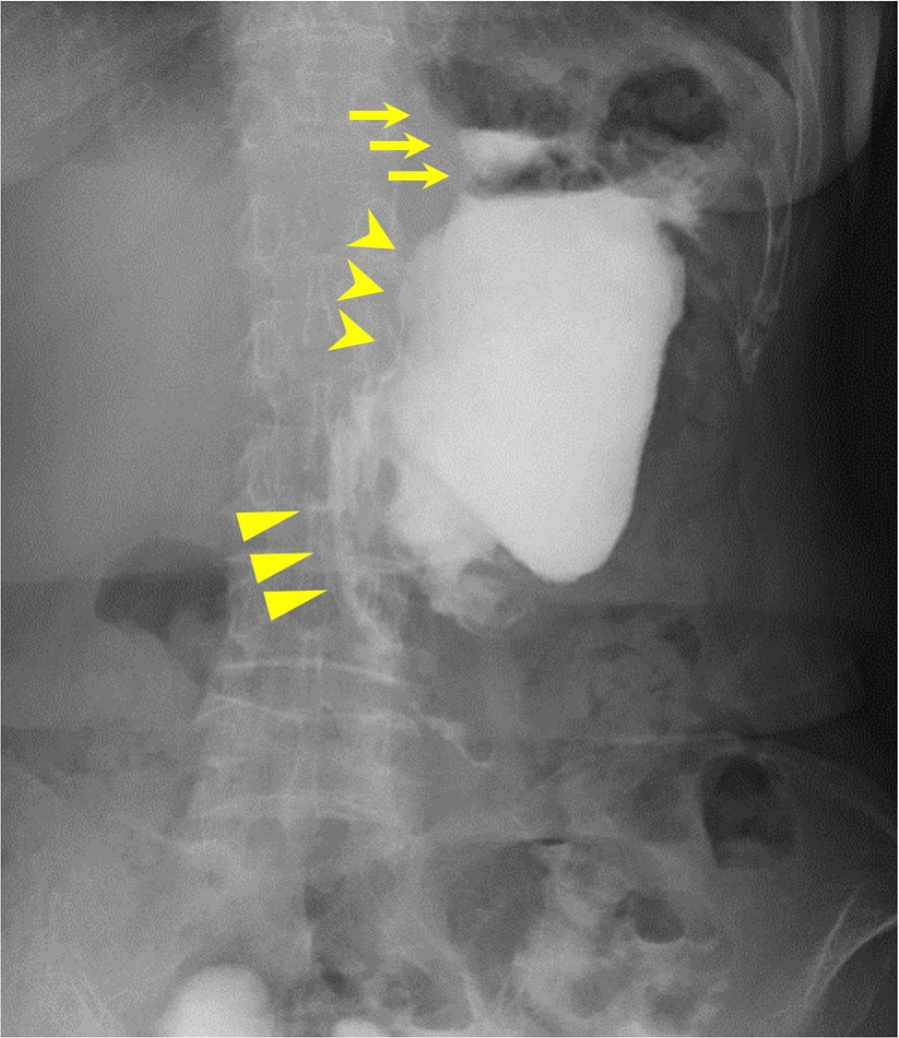
Postoperative oral fluoroscopy with a water-soluble medium. The contrast medium passes from the interposed jejunum to the efferent loop through the remnant stomach. The arrow, arrowhead, and triangle indicate the interposed jejunum, remnant stomach, and efferent loop, respectively

**Fig. 6:**
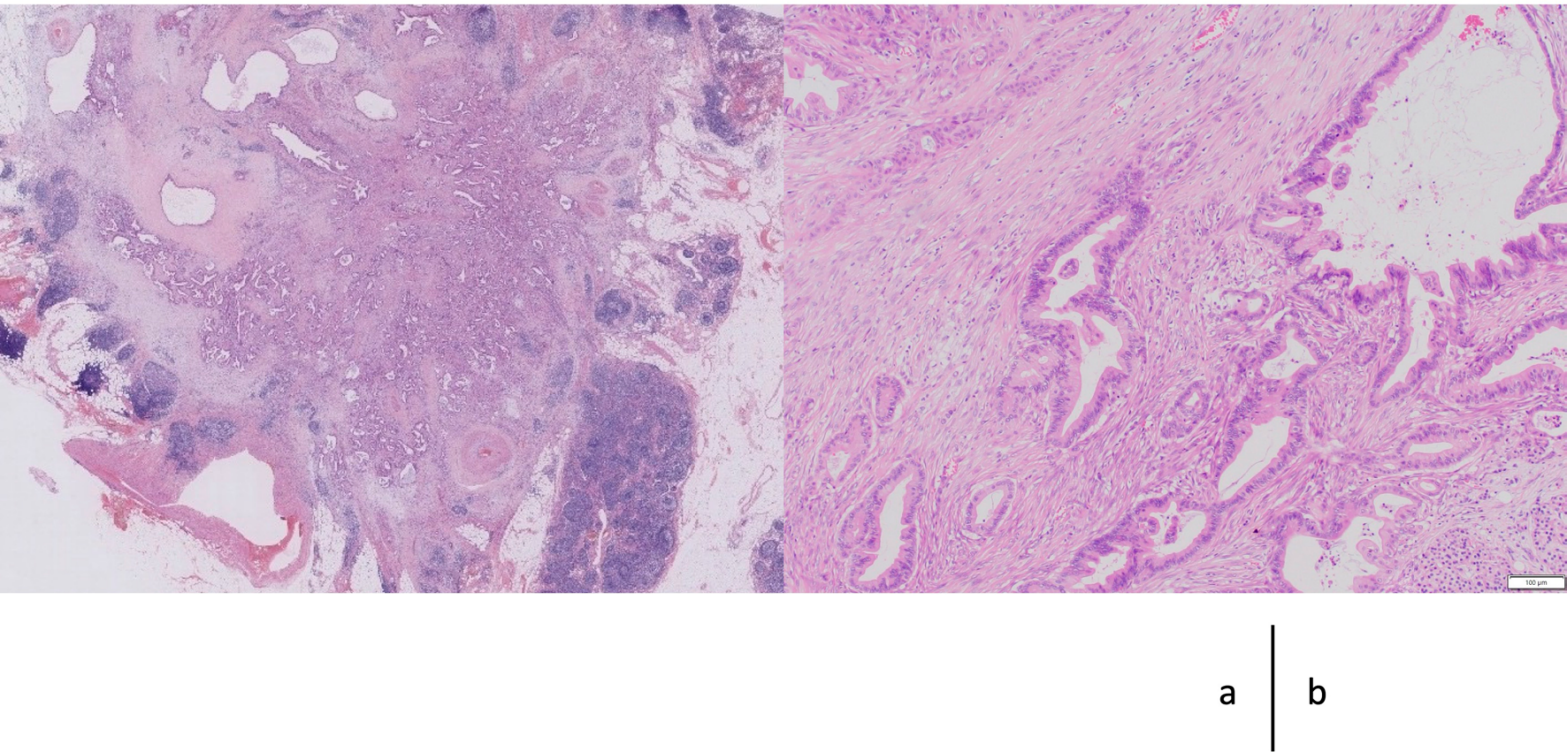
Pathological findings of resected specimen. **a** In the periphery of the invasive carcinoma, cystic glands of intrapapillary mucinous neoplasm are observed. **b** The tumor proliferates showing glandular structure and invades in the fibrotic pancreatic stroma

Postoperatively, the patient developed delayed gastric emptying and aspiration pneumonia. Although intermittent nasogastric tube placement and conservative treatment were necessary, postoperative oral gastrographic imaging revealed smooth flow from the esophagus to the efferent jejunum after recovery (Fig. 5). At 84 postoperative days, she was discharged from our department. Histopathologically, IPMNs were identified in the whole pancreas. Moderately to poorly differentiated tubular adenocarcinoma proliferated and infiltrated in 21 × 15 × 15 mm-sized tumor at the pancreatic head (Fig. 6). Then pathological diagnosis was intraductal papillary mucinous neoplasm with associated invasive carcinoma of the pancreatic head. No lymph node metastases were found. The surgical margin was negative for cancer. Although the patient had lost 7 kg during discharge compared with her preoperative bodyweight, she recovered normal serum albumin levels 4 months postoperatively. Adjuvant chemotherapy with tegafur/gimeracil/oteracil (S-1) was administered from the 5th postoperative month for 6 months. She was followed up in the outpatient clinic for a year and had no recurrence.

## Discussion

Pancreatoduodenectomy with right gastroepiploic conduit preservation or reconstruction is mainly categorized into the following three types: resection following esophagectomy [7–9, 14, 15, 20–22]; resection following coronary artery bypass with the RGEA [5, 10–13, 17, 19, 23], and resection following proximal gastrectomy, as in the current case [7, 16, 18]. Previously, four cases in three literatures on pancreatic head resection with right gastroepiploic conduit preservation or reconstruction following proximal gastrectomy were reported (Table 1). Among them, GDA resection and arterial reconstruction from the GDA to the RGEA anastomosis were performed in one case of pancreatic ductal adenocarcinoma.

**Table 1:** Previous reports of pancreatic head resection following proximal gastrectomy

Author	Year	Age	Sex	Diagnosis	Procedure	RGEV	RGEA	Complication
Ikeda	2001	61	M	Distal cholangiocarcinoma	PD	Preserved	Preserved	Uneventful
Akabane	2016	68	M	Pancreatic cancer	PD	Not described	Reconstructed	Uneventful
Hirashita	2019	73	M	Distal cholangiocarcinoma	PPPD	Preserved	Preserved	Uneventful
Hirashita	2019	58	M	Distal cholangiocarcinoma	PPPD	Preserved	Preserved	Uneventful
Our case	2023	78	F	Pancreatic cancer	TP	Preserved	Preserved	Delayed gastric emptying

*RGEV* right gastroepiploic vein, *RGEA* right gastroepiploic artery, *PD* pancreaticoduodenectomy, *PPPD* pylorus-preserving pancreaticoduodenectomy, *TP* total pancreatectomy

The significance of maintaining the flow of the right gastroepiploic conduit in pancreatectomy depends on the patient’s anatomical background and surgical procedure. In the present case, when the right gastroepiploic conduit was resected, only the right gastric artery and vein remained as vessels of the remnant stomach. Previous studies reported successful remnant stomach preservation in patients undergoing distal pancreatectomy following distal gastrectomy despite few stomach vessels [25–27]. This may be explained by the blood flow from the anastomosed intestinal wall that developed during the interval from the previous gastrectomy, which supports the blood supply to the remnant stomach. Similar to distal gastrectomy, blood supply through the gastrointestinal or esophagogastric anastomosis can be expected to some extent in patients undergoing proximal gastrectomy. However, patients with pancreatic head resection following proximal gastrectomy need new gastrointestinal anastomosis, which is the major difference from those with distal pancreatectomy following distal gastrectomy. Considering that abundant blood flow is important for the proper healing of gastrointestinal anastomosis [28, 29], preserving the right gastroepiploic conduit must have been essential in the present case.

In the present case, the current patient had an interposed jejunum and required total pancreatectomy, which was different from the previous four cases of pancreatoduodenectomy following proximal gastrectomy. To mobilize the pancreatic tail with interposed jejunum preservation, we first encircled the mesenteric stalk lying on the pancreas, and then pulled out the pancreatic tail in front of the mesenteric stalk and remnant stomach. This procedure resulted in a clear the surgical field of vision clear around the roots of the splenic artery and vein, and those vessels could therefore be safely resected. Subsequently, pancreatectomy was completed similar to the standard manner by pulling out the pancreatic body and tail to the right side of the right gastroepiploic conduit. To prevent twisting the blood flow arcade of the jejunal limbs, the reconstruction route of the jejunum following total pancreatectomy must be considered. In our patient, the stalk of the interposed jejunum was approached through the transverse mesentery. The jejunal limb of biliary reconstruction was passed through the retromesenteric route, and the gastrojejunostomy was anastomosed with sequential peristalsis (Fig. 3h). The patient did not develop any ischemic events postoperatively.

In the present case, although the preoperative diagnosis was IPMN, and intraoperative findings did not imply invasion to the GDA and RGEV, the pathological diagnosis was intraductal papillary mucinous neoplasm with associated invasive carcinoma. Considering that the exposed surgical margin and regional lymph nodes were negative for cancer, we did not perform additional resection. However, modified surgeries are generally not suitable for invasive malignancy, and this patient may have undergone standard pancreatectomy with right gastroepiploic conduit resection when she was preoperatively diagnosed with invasive adenocarcinoma.

## Conclusions

We presented a successful case of pancreatectomy with remnant stomach preservation following proximal gastrectomy. To avoid remnant total gastrectomy, right gastroepiploic conduit preservation is an optional procedure of pancreatic head resection in patients who have undergone proximal gastrectomy with reconstruction by jejunal interposition.

## Data Availability

Not applicable.

## Abbreviations

CHA: Common hepatic artery
EUS: Endoscopic ultrasonography
GDA: Gastroduodenal artery
IPMN: Intraductal papillary mucinous neoplasm
RHA: Right hepatic artery
SMA: Superior mesenteric artery
SMV: Superior mesenteric vein
RGEA: Right gastroepiploic artery
RGEV: Right gastroepiploic vein
S-1: Tegafur/gimeracil/oteracil
